# Human β-Defensin 2 Mediated Immune Modulation as Treatment for Experimental Colitis

**DOI:** 10.3389/fimmu.2020.00093

**Published:** 2020-01-31

**Authors:** Louis Koeninger, Nicole S. Armbruster, Karoline Sidelmann Brinch, Søren Kjaerulf, Birgitte Andersen, Carolin Langnau, Stella E. Autenrieth, Dominik Schneidawind, Eduard F. Stange, Nisar P. Malek, Peter Nordkild, Benjamin A. H. Jensen, Jan Wehkamp

**Affiliations:** ^1^Department of Internal Medicine I, University Hospital Tübingen, Tübingen, Germany; ^2^Novozymes, Bagsvaerd, Denmark; ^3^Department of Internal Medicine II, University Hospital Tübingen, Tübingen, Germany; ^4^Defensin Therapeutics, Copenhagen, Denmark; ^5^Department of Medicine, Faculty of Medicine, Cardiology Axis, Quebec Heart and Lung Institute, Laval University, Quebec, QC, Canada; ^6^Section for Human Genomics and Metagenomics in Metabolism, Faculty of Health and Medical Sciences, Novo Nordisk Foundation Center for Basic Metabolic Research, University of Copenhagen, Copenhagen, Denmark

**Keywords:** host defense peptides, antimicrobial peptides, β-defensins, IBD, innate immunity

## Abstract

Defensins represents an integral part of the innate immune system serving to ward off potential pathogens and to protect the intestinal barrier from microbial encroachment. In addition to their antimicrobial activities, defensins in general, and human β-defensin 2 (hBD2) in particular, also exhibit immunomodulatory capabilities. In this report, we assessed the therapeutic efficacy of systemically administered recombinant hBD2 to ameliorate intestinal inflammation in three distinct animal models of inflammatory bowel disease; i.e., chemically induced mucosal injury (DSS), loss of mucosal tolerance (TNBS), and T-cell transfer into immunodeficient recipient mice. Treatment efficacy was confirmed in all tested models, where systemically administered hBD2 mitigated inflammation, improved disease activity index, and hindered colitis-induced body weight loss on par with anti-TNF-α and steroids. Treatment of lipopolysaccharide (LPS)-activated human peripheral blood mononuclear cells with rhBD2 confirmed the immunomodulatory capacity in the circulatory compartment. Subsequent analyzes revealed dendritic cells (DCs) as the main target population. Suppression of LPS-induced inflammation was dependent on chemokine receptor 2 (CCR2) expression. Mechanistically, hBD2 engaged with CCR2 on its DC target cell to decrease NF-κB, and increase CREB phosphorylation, hence curbing inflammation. To our knowledge, this is the first study showing *in vivo* efficacy of a systemically administered defensin in experimental disease.

## Introduction

Inflammatory bowel diseases (IBD) are multifactorial disorders characterized by chronic relapsing inflammation of the intestine (1). They currently affect more than 4 million patients worldwide (2) and are classified in two major entities, Crohn's disease (CD) and ulcerative colitis (UC). While UC is mainly restricted to the colonic mucosa, CD can occur at any site of the gastrointestinal tract but predominantly in the terminal ileum and colon, and inflammation is typically transmural (3).

The etiology of the different forms of IBD is not fully understood. It has, however, been demonstrated that differential defects of the intestinal antimicrobial barrier play an important role in the pathogenesis of both CD and UC (4). Genetic analysis revealed that small intestinal vs. colonic CD are different disease entities and are characterized by distinct but overlapping genetic signatures (5). Moreover, in UC compromised mucus production, due to depletion of goblet cells, is a key triggering event in disease pathology, whereas CD is characterized by a defective intestinal barrier, which associates with complex defensin deficiencies based on a variety of mechanisms (6–10). The best described genetic links to small intestinal CD (11), i.e., NOD2, ATG16L1, XBPD1, are functionally involved in Paneth cell function (12–15). Paneth cells of the small intestine secrete different antimicrobial peptides into the intestinal lumen. Other mechanisms involving compromised α-defensin regulation of Paneth cells include the Wnt signaling pathway (16). A reduced monocyte derived Wnt ligand secretion in CD may further diminish Paneth cells and defensin expression (10). In the colon, we and others have shown an attenuated induction of the inducible human β-defensin 2 (hBD2) in CD patients, although the mechanisms remains elusive (7).

Defensins represent an ancient highly conserved part of the innate immune system. Most of these small endogenous peptides possess broad-spectrum antimicrobial activity as well as immunomodulatory functions. In humans, granulocytes as well as Paneth cells secrete different α-defensins whereas β-defensins are expressed by epithelial surfaces throughout the body (17). hBD2 was discovered using a functional antimicrobial readout by Harder and Schröder in the skin (18). As shown *in vitro* hBD2 has strong antimicrobial and immunomodulatory functions and is induced by inflammatory stimuli or exogenous microbial substances (19). hBD2 promotes intestinal wound healing (19) and angiogenesis (20) *in vitro* and can act as a chemoattractant for dendritic cells (DCs), monocytes and T-cells through interaction with the chemokine receptor 2 (CCR2) and 6 (CCR6) (21, 22). Thus, in addition to a lack of mucosal antibacterial activity (23) low defensin expression may also translate into a repressed anti-inflammatory activity. Together, these data provide evidence for an important role of defensins, including hBD2, in IBD disease pathogenesis and potential therapy, but its mode of action *in vivo* and their potential role as therapeutics remains to be described.

Standard therapy in IBD is based on immunosuppression with glucocorticosteroids and azathioprine as short and long term therapy, respectively. Antibodies that target tumor necrosis factor alpha (TNFα) attenuate disease-related inflammatory pathways rather than act as a general immunosuppressants, but 20–40% of patients are primary TNFα non-responders and up to 50% lose their effective response over time, termed secondary non-responders (24, 25). Despite successful development of other biologicals against specific targets like integrins or IL-12/23, the medical need for alternative therapeutic strategies targeting the molecular mechanisms underlying IBD is still high, providing a sound rationale for examining hBD2 as a potential biological therapy for the treatment of IBD and potentially other barrier function related inflammatory disorders. However, a major limitation for considering development of hBD2 was the difficulty to produce sufficient quantities of defensin peptides at industrial scale. We have therefore developed a cost efficient large-scale production method of recombinant hBD2 (26). In this study, we hypothesized that hBD2 could act as an anti-inflammatory peptide independently of its classical antimicrobial function. We found that recombinant hBD2 suppressed DC-mediated secretion of proinflammatory cytokines such as TNF-α, IL-12 and IL-1β. The mechanism was dependent on CCR2 signaling leading to a reduced NF-κB but increased CREB phosphorylation. Extending the *in vitro* findings, we next assessed the capability of hBD2 to suppress IBD in three different animal models of experimental colitis. We administered the therapeutic agent by subcutaneous injections to uncouple its classical antimicrobial actions from its immunomodulatory capabilities. hBD2 administration significantly improved the responding phenotype in both DSS-, TNBS-, and T-cell induced colitis, hence corroborating broad treatment efficacy in discrepant gastrointestinal disease pathologies. These data represent the first *in vivo* evidence that a human defensin, such as hBD2, offers a systemic, anti-inflammatory biologic agent, which could be used as a promising future therapeutic against human IBD.

## Materials and Methods

### Human Blood Samples

In this study blood was obtained from healthy individuals (males and females in 1:1 ratio) that gave their written and informed consent after they were informed about the study purpose, sample procedure, and potential adjunctive risks. The study protocol was previously approved by the Ethical Committee of the University Hospital, Tübingen, Germany and the Ethical Committee of Region Capital, Denmark (Den Videnskabsetiske komite Region Hovedstaden).

### Production and Purification of Recombinant hBD2

Recombinant hBD2 was expressed in *E. coli* as a his-tagged thioredoxin fusion protein with an enterokinase cleavage site and purified essentially as described in the patent (WO2010/007166 Treatment of inflammatory bowel diseases with human β-defensin 2). An additional reversed phase purification step was included to ensure removal of endotoxins. The processed and purified hBD2 was diluted in water for injection supplemented with 1% v/v formic acid and bound to a Daisogel SP-120-C18 column and eluted with 1% v/v formic acid in 30% v/v ethanol. The solvents were removed by evaporation in a speed-vac and the final product formulated in PBS before use. The proper folding and disulphide-bridge topology was verified using tryptic digestion coupled with LC-MS/MS and NMR spectroscopy. The purified hBD2 (endotoxin levels <0.05 EU/ml) were kept in its natural tertiary structure with purity ≥96%.

### *In vitro* Toxicity Tests

#### Red Blood Cell Assay

Blood was collected using EDTA as anticoagulant and diluted in PBS to obtain an 8% red blood cell suspension. One part of the red blood cell suspension was added to three parts of test material (dissolved in PBS) in a poly propylene plate. One percent sodium dodecyl sulfate (SDS) was used as a positive control. Assay mixtures were incubated for 60 min at 37°C under constant agitation of the plate. Incubation was terminated by centrifugation at 2,000 rpm for 2–3 min. 50 μl of the supernatants were transferred to a microtiter plate and measured at 540 nm.

#### Murine Fibroblasts L929 Neutral Red Uptake

Cytotoxicity was measured by the neutral red uptake procedure of Borenfreund and Puerner, using mouse L929 fibroblasts (ATCC® CCL-1^TM^) and a 24 h exposure to hBD2 (27). L929 fibroblasts were grown in EMEM supplemented with 10% fetal bovine serum (FBS). For testing 7.5 × 10^4^ cells were seeded into 96 well plates and incubated for 24 h at 37°C to establish a near confluent monolayer. Cells were challenged with the indicated concentrations of hBD2, SDS was used as control.

### *In vivo* Toxicity Test

*In vivo* toxicity of hBD2 was assessed in 6–8 weeks old female NMRI mice (Taconic Europe). All animal studies were performed according to Danish legislations for laboratory animals and approved by Novozymes science ethics committee. hBD2 was given subcutaneously (s.c.) in the intrascapular region using a 25G needle and a 1 ml syringe. Animals were dosed on day 0 with the indicated amount of hBD2, applied as 10 mg/kg according to the individual body weight. Clinical signs were recorded on day 0 and monitored until experimental endpoints. Body weights were recorded as a minimum on day 0, day 2, and day 4 prior to euthanasia. Necropsy was performed after euthanasia and kidneys, spleens and livers were weighed.

### Pharmacokinetic Profile of hBD2

Two groups of female NMRI mice were weighed and injected s.c. with either 1 mg/kg (*n* = 4) or 10 mg/kg (*n* = 3) hBD2 in 300 μl. HBD2 was diluted in 10 mM sodium acetate in 0.9% NaCl. Blood samples were collected at different time points and stored at room temperature for a minimum of 20 min before centrifugation at 2,000 × g for 10 min. Serum was separated and stored at −20°C until analysis. Serum from the group that received 10 mg/kg was analyzed by LC-MS/MS, the serum from the other group was analyzed by HPLC.

### Stimulation of Peripheral Blood Mononuclear Cells (PBMCs)

**Figure 1:**
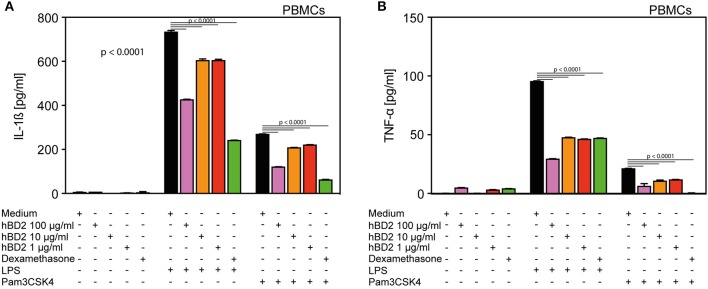
hBD2 reduced the pro-inflammatory effect of LPS and Pam3CSK4 in human primary PBMC's. PBMC's were challenged with LPS (20 pg/ml) or Pam3CSK4 (0.3 μg/ml), respectively, and treated with various concentrations of hBD2 (100, 40, 10, or 1 μg/ml). Release of **(A)** IL-1β and **(B)** TNF-α. Results are presented as mean ± SEM, **(A)** and **(B)** *n* = 150–194. Statistical analysis was performed by one-way ANOVA with Bonferroni post-test.

**Figure 2:**
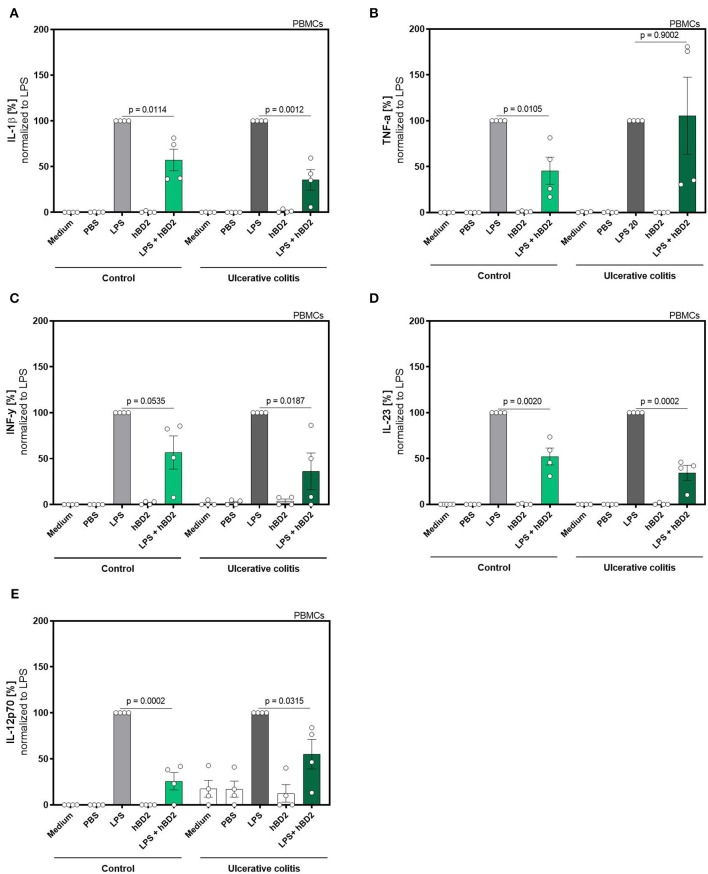
hBD2 reduced the pro-inflammatory effect of LPS in human primary PBMC's of ulcerative colitis patients. PBMC's were challenged with LPS (20 pg/ml) and treated with 10 μg/mL hBD2. Release of of **(A)** IL-1β, **(B)** TNF-α, **(C)** INF-y, **(D)** IL-23, and **(E)** IL-12p70 is shown in % normalized to LPS. Data are presented as mean ± SEM (*n* = 4) and analyzed by unpaired *t*-test.

Heparinized blood was diluted 1/1 v/v with RPMI (Gibco) and was subjected to Ficoll-Paque Plus (GE healthcare) density centrifugation within 2 h of drawing. Plasma was collected from the top from individual donors and was kept on ice until it was used at 2% in the culture medium (autologous culture medium). Isolated PBMC's were re-suspended in autologous culture medium and seeded in 96-well culture plates with 115.500 cells per well (Figure 1) or 200.000 cells per well (Figure 2) in a total of 200 μl. PBMC's from the same donor were stimulated with 100, 10 or 1 μg/ml of hBD2 either alone or together with 20 pg/ml lipopolysaccharide (LPS) (*E. coli*, O111:B4, Sigma L4391) or with 0.3 μg/ml Pam3CSK4 (InvivoGen). The supernatants were collected after incubation at 37°C for 24 h, and stored at −80°C until cytokine measurement.

The experiment in Figure 1 was performed with healthy volunteers, which were recruited under an approval from the Ethical Committee for Region Capital, Denmark. Interleukin 1β (IL-1β) and tumor necrosis factor alpha (TNF-α) (Figure 1) were quantified in supernatants by flow cytometry using a human inflammation cytometric bead array (CBA) according to manufacturer's instructions (BD) using a FACSarray flow cytometer. IL-1β, TNF- α, Interferon gamma (INF-y), Interleukin 12p70 (IL-12), and Interleukin 23 (IL-23) were analyzed using a human inflammation cytometric bead array (LegendPlex Biolegends) according to manufacturer's instructions.

The experiment in Figure 2 was performed with 8 subjects (4 controls and 4 colitis patients) to study the impact of disease status on treatment efficacy. Clinical status of these subjects were subtotal remission (Simponi and Salofalk), remission (Vedolizumab), mild disease (Infliximab, Salofalk, and Prednisolon), and moderate disease (Vedolizumab). PBMCs from these subjects were analyzed for IL-1b, TNF-a, IFN-g, IL-12, and IL-23 expression using a human inflammation cytometric bead array (LegendPlex Biolegends) according to manufacturer's instructions.

### Generation of Human Monocyte-Derived Dendritic Cells (Mo-DCs)

Peripheral blood was drawn from healthy donors and diluted one to one in PBS. The mixture was stacked in a falcon tube on Biocoll separation solution (Biochrome) with a ratio of three parts blood PBS mixture to two parts Biocoll. For a density gradient centrifugation the falcon tubes were centrifuged at 2,000 rpm for 30 min at RT. The PBMC's were removed and washed twice with PBS. 1.5 × 10^6^ cells were seeded per well in a tissue culture-treated 6-well plate in RPMI-1640 medium (Merck) supplemented with 10% fetal calf serum (FCS; Sigma-Aldrich), 2 mM L-glutamine (Biochrome), 100 U/ml penicillin/streptomycin (Gibco), 50 μM 2-mercaptoethanol (Fluka), 1 mM sodium pyruvate (Biochrome), and 1x non-essential amino acids (Biochrome) and incubated for 1 h at 37°C and 5% CO_2_ for monocyte adherence. Then the cells were washed with media and PBS to remove the non-adherent cells and were cultivated for 6 days with media additional supplemented with 50 ng IL-4 and 100 ng GM-CSF (Miltenyi). Cytokines were added a second and third time on day 2 and 4 whereas the cells were harvested at day 6.

### Generation of Bone Marrow-Derived Dendritic Cells (BM-DCs)

BM-DC's were generated using granulocyte-macrophage colony-stimulating factor (GM-CSF) and RPMI-1640 medium (Merck) supplemented with 10% fetal calf serum (FBS; Life Technologies), 2 mM glutamine (Thermo Fisher), 100 U/ml penicillin/streptomycin (Gibco), 50 μM 2-mercaptoethanol (Roth), 1 mM sodium pyruvate (Biochrome), and 1x non-essential amino acids (Biochrome) as previously described (28). Shortly, 2 × 10^6^ bone marrow cells flushed from the tibias and femurs of C57BL/6 mice were seeded in dishes containing 200 U/ml GM-CSF. After 3 days extra medium containing GM-CSF was added to the cells and on day 6 half of the medium was replaced by fresh serum containing GM-CSF. After 7 or 8 days slightly attached cells were harvested. Female C57BL/6JolaHsd mice were purchased from Janvier (St. Berthevin Cedex, France). Animal experiments were performed in strict accordance with the German regulations of the Society for Laboratory Animal Science (GV-SOLAS) and the European Health Law of the Federation of Laboratory Animal Science Associations (FELASA). The protocol was approved by the Regierungspräsidium Tübingen (Anzeige 09.01.2014).

### Cytokine Production by Human Mo-DC's and Murine BM-DC's

2 × 10^5^ murine BM-DC's or human Mo-DC's were seeded in 96-well round bottom plates. First they were pretreated with 100 ng/ml pertussis toxin (Sigma-Aldrich) or 5 μM of the CCR2 inhibitor RS 504393 (Tocris) and subsequently stimulated with 100 ng/ml LPS or 0.2 mg/ml TNF-α, 0.2 mg/ml IL-6 and 0.2 mg/ml IL-1β and 10 μg/ml or 100 μg/ml hBD2. 24 h later supernatants were collected and TNF-α [Biolegend (BM-DC's); R&D (Mo-DC's)] was analyzed according to the manufacturer's instructions. IL-10, IL-12, and IL-1β (LEGENDplex Biolegend) was as well-analyzed according to the manufacturer's instructions.

### Flow Cytometry

2 × 10^5^ murine BM-DC's were seeded in 96-well round bottom plates and treated as described above. Cells were removed from the plate using Accutase (Sigma-Aldrich) and stained for 20 min at room temperature with Zombie Aqua (Biolegend) to exclude dead cells and extracellular antibodies against CD11c-APC (N418) (Miltenyi), MHCII-FITC (M5/114.15.2) (Miltenyi) and CD86-BV421 (GL-1) (Biolegend). For p-CREB staining cells were fixed and permeabilized with Foxp3 Staining Buffer Set (eBioscience) and stained with primary antibody phosphor-CREB mAb (Ser133; clone 87G3) (Cell Signaling) for 30 min in the dark at room temperature followed by secondary goat anti-rabbit IgG-DyLight™649 (Jackson ImmunoResearch) for 15 min at 4°C. To detect intracellular p-NF-κB BM-DCs were fixed with 2% paraformaldehyde (VWR) in PBS, permeabilized with 90% freezing methanol (Applichem) and stained with the primary antibodies to phosphor-NF-κB p65 (93H1) (Cell Signaling) for 60 min in the dark at room temperature followed by goat anti-rabbit IgG-PE-Cy7 (Santa Cruz Biotechnology) for 15 min at 4°C. PBS with 0,5% bovine serum albumin (Biomol) was used for all incubations and washing steps. At least 50,000 cells were acquired using a Canto-II flow cytometer (BD nces) with DIVA software (BD Biosciences) and were further analyzed using FlowJo 10.5 software (Tree Star).

### DSS Colitis Model

The DSS colitis study was performed by Farma-Cros Ibérica according to directive 86/609 EEC and approved by Novozymes science ethics committee. 7–8 weeks old male C57BL/6 mice were used (Charles River) and each group consisted of 10 animals. Animal allocation to all experimental groups was done in a randomized manner. Colitis was induced by supplementing the drinking water with 2% dextran sodium sulfate (DSS, 30–50 kDA, MP Biomedicals) for 7 days. On day 1 all mice were weighed and the drinking bottle was filled with the DSS solution, this solution was replaced on day 3 and 5. On day 8 the remaining solution was discarded and replaced with autoclaved water. Mice were divided into 3 groups. One group received PBS as sham treatment intravenously. One group received on day 1, 4, and 8, 300 μg/mouse of a mouse anti-TNF-α antibody (αTNF, Ramcon) intraperitoneally. The animals in the other group were dosed s.c. with 0.1 mg/kg hBD2 once a day, starting at day 1 until day 10. Animals were euthenized on day 10. Daily clinical assessment was carried out to calculate a validated clinical disease activity index (DAI) ranging from 0 to 4 according to the following parameters: body weight loss, presence or absence of rectal bleeding, stool consistency. One mouse in the DSS control group had to be euthanized before the end of the study.

### TNBS Colitis Model

The TNBS colitis study was performed by Farma-Cros Ibérica according to directive 86/609 EEC and approved by Novozymes science ethics committee. Male BALB/cByJ mice were used (Janvier) and each group consisted of 15 animals. Animal allocation to all experimental groups was done in a randomized manner. Colitis was induced on day 0 by intracolonic (distal) administration of trinitrobenzene sulfonic acid (TNBS), 1 mg/mouse in 50% ethanol under mild anesthesia (ketamine/xylazine). Treatment of the animals started on day 0 after induction of colitis. All compounds were applied s.c. Mice received PBS (TNBS control), Prednisolone (10 mg/kg), or hBD2 0.1 mg/kg), respectively. In the TNBS control group as well as in the Prednisolone group, 2 animals had to be sacrificed before the end of the experiment. All other animals were sacrificed on day 10. Daily clinical assessment was carried out to calculate a validated clinical DAI ranging from 0 to 4 according to the following parameters: body weight loss, presence or absence of rectal bleeding, stool consistency.

### T Cell Colitis Model

This study was performed by the Department of Biomedical Science at the University of Catania according to directive 86/609 EEC and approved by Novozymes science ethics committee. 8 weeks old female BALB/c and C.B-17 female SCID mice were purchased from Harlan (Italy). Colitis was induced in severe combined immunodeficiency (SCID) mice by transplantation of CD4^+^/CD25^−^ T cells from the BALB/c mice. Briefly, lymphomonocytes isolated from spleen or lymph nodes from BALB/c mice were subjected to negative selection of CD4^+^ T cells. Afterwards, CD4^+^/CD25^+^ cells were positively isolated by binding to the beads from the CD4^+^ T cell suspensions and the CD4^+^/CD25^−^ were collected from supernatant. Cell preparation was considered successful if the analysis of purified cells by flow cytometry (FACSCalibur, BD Bioscience, Heidelberg, Germany) using CellQuest software showed that >95% of the cells were viable (based on forward and side-scatter characteristics and/or 7- actynomycin-D staining) and CD4-positive (using a FITC-conjugated anti-mouse CD4-antibody, BD, Heidelberg, Germany), as well as more than 98% depleted of CD25 (using a APC-conjugated anti-mouse CD25-antibody, BD, Heidelberg, Germany). CD4^+^/CD25^−^ cells were intraperitoneally injected to SCID mice at a concentration of 500,000 cells in a final volume of 0.2 ml RPMI 1640. Sham treated animals (*n* = 6) received 0.2 ml pure RPMI 1640. Diseased mice were randomized divided into 4 groups (*n* = 11) and treated once daily by s.c. application of PBS (vehicle), hBD2 (0.1 and 1 mg/kg, respectively) or 0.3 mg/kg Dexamethasone (Dexa., applied intraperitoneally). Treatments started 7 days post T cell transfer and continued daily for 86 days. Animals were weighed three times a week and monitored twice a week starting from day 42 for the clinical status, summarized as DAI. The DAI included body weight loss, stool consistency, and the presence of blood at the rectum. One animal in the vehicle and one in the Dexamethasone treated groups had to be sacrificed before the end of the study. At the end of the study, animals were sacrificed and the colon was removed and carefully cleaned for further analysis. First, the colon was weighed then a section from the middle was removed and fixed for pathologic survey. The rest of the colon (5 cm) was used to quantify the activity of myeloperoxidase in the tissue.

### Myeloperoxidase Activity Assay

Myeloperoxidase activity was performed on 5 cm of colon previously frozen at −80°C. Colon was homogenized in 0.5% of HETAB dissolved in 10 mM of Phosphate-Citrate Buffer (pH 7.0) to enable the release of MPO enzyme from the neutrophil granules. Homogenated samples after three freeze-thaw cycles were spun at 3,000 × g at 4°C for 30 min. Pellets were resuspended in 0.5% HETAB in 10 mM Phosphate-Citrate Buffer and spun again at 3,000 × g at 4°C for 30 min. 500 ul of supernatant were delivered into a vial along with 500 ul of TMB in Phosphate-Citrate Buffer containing Perborate Sodium. Changes in absorbance at 620 and 450 nm were read by a spectrophotometer (IRIS). Peroxidase enzyme diluted in 0.5% HETAB in 10 mM Phosphate-Citrate Buffer and H2O was used as standard. Two-fold dilutions of standard were prepared at the highest concentration of 50 pg/ml. Reagents and samples were kept refrigerated. Substrate solution was prepared just before the assay. MPO activity expresses the amount of enzyme that is able to degrade 1 μM of peroxide/min and it is expressed as U/g tissue weight.

### Preparation of Histological Samples

Sections of the colon were taken from each animal and preserved in neutral buffered formaline for subsequent histological analysis. Sections were stained using haematoxylin and eosin for further scoring.

### Statistical Analysis

Release of cytokines from PBMC's was compared using two-way ANOVA and Tukey post-test or unpaired student's *t*-test as appropriate. Weight change, colon weight and MPO activity were compared using one-way-ANOVA and Bonferroni post-test, while clinical and pathological scores were analyzed by a Kruskal-Wallis-test for non-parametric data with a Dunn's post-test. All statistical analyses and graphs were done using GraphPad Prism 8 (GraphPad Software, USA).

## Results

### hBD2 Modulated the Effects of Toll-Like Receptor Ligands in PBMC's

We selected human PBMCs challenged with LPS as an *ex vivo* read out model to assess the anti-inflammatory potential of hBD2. While LPS-challenged PBMCs secreted ample amount of interleukin 1-β (IL-1β) (Figure 1A) and TNF-α (Figure 1B), co-treatment with increasing doses of hBD2 consistently mitigated release of both cytokines. hBD2 is a positively charged peptide and could potentially bind to the negatively charged LPS, hence mediate the observed effect indirectly without interfering with the pathway. To investigate this possibility, we included an alternative toll like receptor (TLR) ligand, Pam3CSK4; a neutral/negative charged synthetic peptide. As shown in Figure 1, hBD2 significantly reduced the Pam3CSK4 induced release of IL-1β and TNF-α, corroborating a strong anti-inflammatory effect of hBD2 on human PBMCs *ex vivo*.

We next evaluated if the immune modulating capabilities of hBD2 could be extended to colitis patients. This patient group is known to exhibit an altered response to immunomodulatory stimuli compared to healthy controls. We therefore expanded the proinflammatory panel to also include interferon (INF)-**γ**, IL-12p70, and IL-23. Despite the low n-size in this proof-of-concept sub-experiment, hBD2 treatment successfully attenuated the proinflammatory immune response in LPS-stimulated PBMCs (Figure 2).

### Cytokine Production of DC's Was Affected by hBD2 in a TLR- and CCR2-Dependent Manner

**Figure 3:**
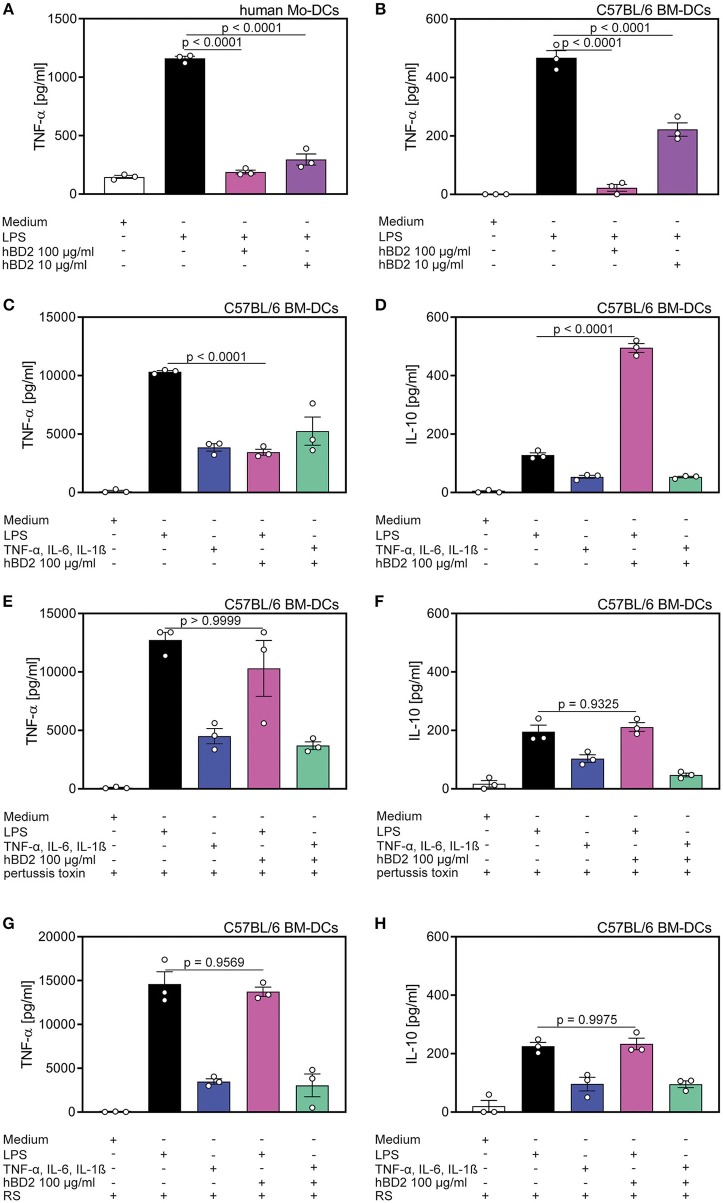
Cytokine production of DC's was affected by hBD2 in a TLR- and CCR2-dependent manner. Human mo-DC's and murine BM-DC's were treated with LPS (10 μg/ml) alone or co-incubated with various concentrations of hBD2 (100 μg/ml or 10 μg/ml). Murine BM-DCs were additionally treated with pertussis toxin or the CCR2 inhibitor RS prior to stimulation with LPS or a cytokine cocktail containing TNF-α (0.2 mg/ml), IL-6 (0.2 mg/ml), and IL-1β (0.2 mg/ml). Release of TNF-α in **(A)** human Mo-DC's and in **(B)** murine BM-DC's was quantified by ELISA. Release of TNF-α **(C,E,G)** and IL-10 **(D,F,H)** in murine BM-DCs was quantified by LEGENDplex. Results are presented as mean ± SEM, *n* = 3. Statistical test used is one-way ANOVA with Bonferroni post-test.

Since PBMCs comprise a variety of cell types we aimed to characterize the responsive cell type, and furthermore the receptor that was targeted by hBD2. DCs upregulate cytokine production when their TLRs engage their cognate ligands (29). Thus, we hypothesized that DC mediated cytokine secretion was affected by hBD2. We generated human mo-DCs and murine BM-DCs *in vitro*, and subsequently challenged them with LPS with or without two different concentrations of hBD2. Similar to the previously reported PBMC response (Figures 1, 2), hBD2 treatment dose-dependently curbed TNF-α production in LPS-challenged DCs (Figures 3A,B).

To test whether the hBD2-mediated cytokine modulation was restricted to TLR signaling, we next investigated the cytokine production of BM-DCs stimulated with a TLR-independent cytokine cocktail. While hBD2 treatment alleviated LPS-induced TNF-α, IL-12p70, and IL-1β secretion concomitant with a substantial induction of the anti-inflammatory cytokine, IL-10, same treatment failed to modulate TLR-independent activation of BM-DCs (Figures 3C,D and Figures S1B,C).

Since hBD2 is able to bind to G protein-coupled receptors expressed on monocytes and DCs (22), we pretreated BM-DCs with pertussis toxin prior to the stimulation with LPS and hBD2. Inhibition of G protein-coupled receptor signaling prevented the observed immunomodulatory capacity of hBD2. Thus, BM-DCs pre-treated with pertussis toxin and stimulated with LPS and hBD2 showed similar TNF-α, IL-1β, IL-10, and IL-12p70 secretion compared to BM-DCs treated solely with LPS (Figures 3E,F and Figures S1D,E).

To specify the G protein-coupled receptor interaction of hBD2 we pre-treated BM-DCs with the CCR2 specific inhibitor, RS504393, and subsequently stimulated with LPS and hBD2. Pre-treatment with RS504393 prevented the anti-inflammatory effect of hBD2 (Figures 3G,H and Figures S1F,G). Together these data demonstrate a central involvement of CCR2 in hBD2-mediated DC cytokine modulation; a trait that was shared between human (Figure S1A) and mouse DCs.

### hBD2 Modulates NF- κB and CREB Phosphorylation

**Figure 4:**
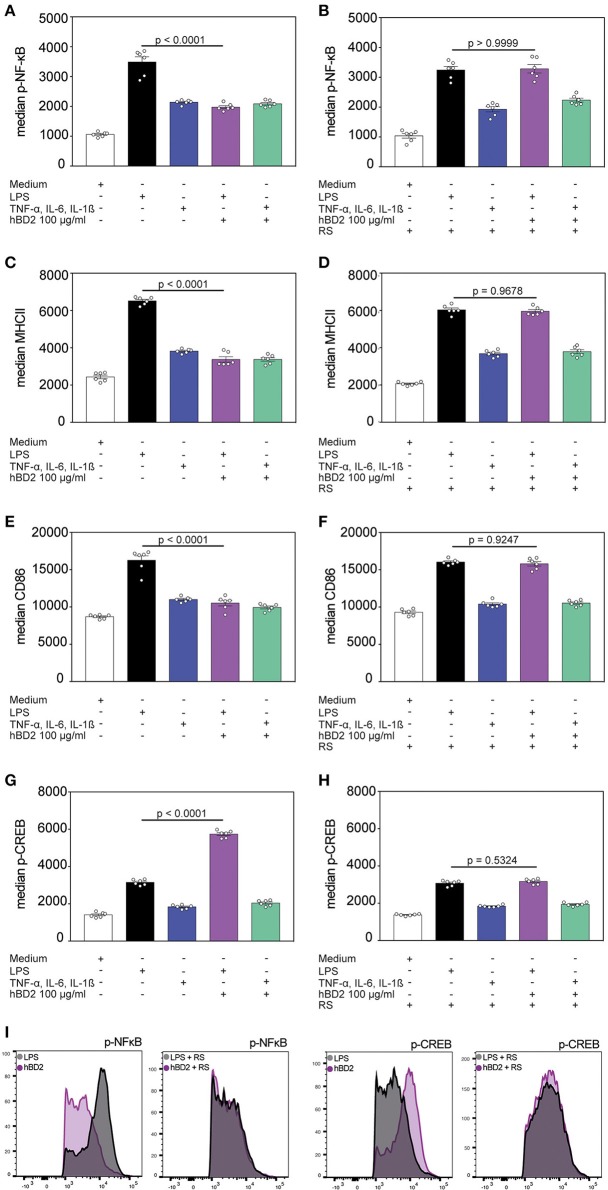
hBD2 modulates NF-κB and CREB phosphorylation. BM-DC's were incubated for 60 min with LPS (10 μg/ml) or a cytokine cocktail containing TNF-α (0.2 mg/ml), IL-6 (0.2 mg/ml), and IL-1β (0.2 mg/ml) alone or in combination with hBD2 (100 μg/ml). BM-DCs were additionally pretreated with pertussis toxin or the CCR2 inhibitor RS prior to stimulation. The cells were stained with CD11c, MHCII, and CD86 antibodies followed by intracellular staining against p-NF-κB or p-CREB and analysis by flow cytometry. Statistical analysis of **(A,B)** p-NF-κB, **(C,D)** MHCII, **(E,F)** CD86, and **(G,H)** p-CREB staining. **(I)** Shows histograms of FACS analysis. Results are presented as mean ± SEM, *n* = 6. Statistical test used is one-way ANOVA with Bonferroni post-test.

NF-κB represents a key signaling pathway triggered by TLRs. This pathway can also be activated by the proinflammatory cytokines TNF-α and IL-1β (30). NF-κB activity is mediated by direct interaction with the CREB coactivator CBP. However, phosphorylated CREB, that can be induced by G protein-coupled receptors, needs in addition CBP to compete with NF-κB and thereby limiting the NF-κB activity (31). We analyzed the NF-κB and CREB phosphorylation of hBD2 treated BM-DCs. Stimulation with proinflammatory cytokines showed an increase in NF-κB phosphorylation although to a lower extent than what was observed during LPS stimulation. Co-incubation of both stimuli with hBD2 showed only a reduced NF-κB phosphorylation when BM-DCs were treated with the TLR ligand LPS in combination with hBD2 (Figures 4A,I). However, pre-treatment with the CCR2 inhibitor RS504393 prior to stimulation showed comparable levels of NF-κB phosphorylation irrespective of hBD2 treatment (Figures 4B,I). CCR2 inhibition therefore prevented the hBD2-mediated reductions in NF-κB activation. Although the mechanism behind this observation remains elusive, LPS-challenged BM-DCs showed reduced maturation status when stimulated with hBD2, corroborated by lower levels of their key activation markers, MHCII and CD86 (Figures 4C,E). Again, pre-treatment with the CCR2 inhibitor prevented this lower expression (Figures 4D,F). We hypothesized that the CREB signaling pathway could counter NF-κB phosphorylation. We therefore analyzed the CREB phosphorylation status in a next step of experiments. We demonstrated that triggering G protein-coupled receptors led to an increase in CREB phosphorylation (Figures 4G,I) and simultaneously to a reduced NF-κB phosphorylation (Figure 4A), suggesting that phosphorylated CREB competes with NF-κB for their mutual cofactor, CBP. In contrast, pre-treatment with the CCR2 inhibitor prevented the increase in CREB phosphorylation (Figures 4H,I).

### Recombinant hBD2 Showed Good Tolerability and Rapidly Entered the Bloodstream After Subcutaneous Administration, Hence Allowing Systemic Immunomodulatory Actions

To identify possible toxicity of recombinant expressed hBD2 we first addressed the hemolytic potential of hBD2 on human red blood cells. As shown in Figure S2A we could not detect any hemolytic effect of ≤300 μg/ml hBD2. We next tested the effect on the viability of murine fibroblasts. No negative effect of hBD2 up to a concentration of 1750 μg/ml could be identified (Figure S2B). Combined, these data corroborates high *in vitro* tolerability. To assess hBD2 toxicity *in vivo*, NMRI mice were challenged s.c. with different doses of hBD2 and monitored for 4 days for the development of clinical symptoms. On day 4, mice were euthanized for necropsy. Table 1 records the clinical symptoms observed. Although minor acute effects were observed (minutes), no adverse effects were observed at necropsy. Shortly after challenge (10–15 min) the mice receiving 10 or 40 mg/kg hBD2 showed mild clinical signs such as decreased locomotor activity, proneness, ptosis, piloerection, pruritus, bradypnoea, reddish discoloration around the eyes and swelling around the eyes and snout. All clinical signs were transient and only mice dosed with 40 mg/kg hBD2 were still affected at 60 min post dosing. Of note, 40 mg/kg is 40 times higher than the highest dose used in our *in vivo* experiments. The lowest dose group, 0.5 mg/kg hBD2 and the vehicle group did not develop any clinical signs after systemic challenge. No further clinical signs were observed during the subsequent 4 day observation period nor could we find any abnormalities during necropsy. Additionally, body weight gain and organ weight were recorded. Mice did not show any weight loss nor did the organ weight differ significantly between the groups (Figures S2C,D). In summary, these results indicate that hBD2 is well-tolerated *in vivo*.

**Table 1:** Clinical symptoms after subcutaneous administration of hBD2.

**Test compound**	**Dose level mg/kg**	**Clinical signs on the day of dosing**				**Necropsy**
		**No clinical signs**	**Pruritus**	**Clinical signs**	**Clinical signs at the end of the day of dosing**	**Macroscopic findings**
Vehicle	0	3/3	0/3	0/3	0/3	0/3
HBD2	0.5	3/3	0/3	0/3	0/3	0/3
	10	0/3	3/3	3/3	0/3	0/3
	40	0/3	3/3	3(severe)/3	0/3	0/3

We next analyzed whether s.c. administered hBD2 would enter the blood stream. For that purpose we injected mice s.c. with 1 or 10 mg/kg hBD2 and quantified the amount of hBD2 in serum at different time points after injection. As shown in Figure S2E, hBD2 entered the blood stream rapidly after s.c. injection and remained detectable for more than 2 h. These findings indicate that s.c. applied hBD2 might not only act locally but could also have systemic effects *in vivo*.

### hBD2 Ameliorated the Outcome of DSS Colitis *in vivo*

**Figure 5:**
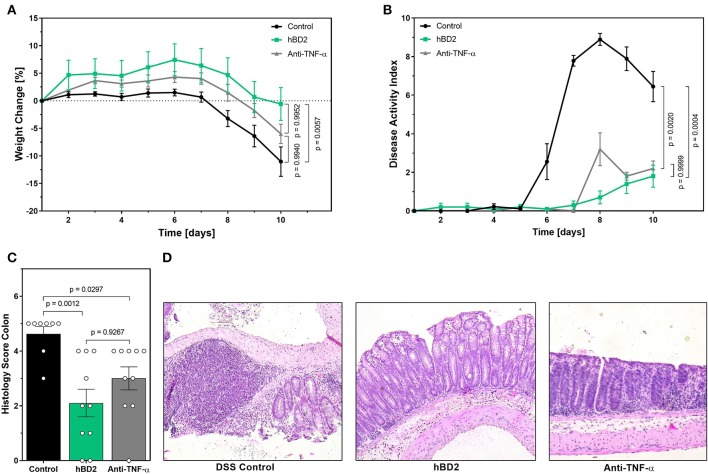
hBD2 ameliorated the outcome of DSS colitis *in vivo*. Colitis was induced by adding 2% DSS into the drinking water. On day 8 DSS was removed from the drinking water. Mice were treated either once a day s.c. with 0.1 mg/kg hBD2 or intraperitoneally with an anti-TNFα antibody (300 μg/mouse) on day 1, 4, and 8. **(A)** Weight change of mice during the experiment, **(B)** development of DAI, **(C)** histology score from the colon of the mice at the end of the experiment, and **(D)** representative images from the colon of differentially treated mice. Results are presented as mean ± SEM, control group *n* = 9 and treated groups *n* = 10. Appropriate statistical comparison are shown within the graph by a Kruskal-Wallis-test for non-parametric data with a Dunn's post-test.

The above described results prompted us to investigate the clinical potential of hBD2 using murine models of experimental colitis with different disease pathologies. First, we assessed the anti-inflammatory and protective effect of hBD2 in DSS colitis. DSS causes a chemical injury to the intestinal mucosa. This results in the exposure of the lamina propria and submucosal compartments to luminal antigens and enteric bacteria which results in inflammation and ulcer formation (32). Treatment of DSS colitis with s.c. administered hBD2 (0.1 mg/kg) resulted in a significant improvement of colitis; the therapeutic effect was superior to anti-TNF-α treatment (Figure 5). hBD2 prevented excessive weight loss (Figure 5A) and improved the DAI (Figure 4B). Furthermore, scoring of the colonic mucosa for damage revealed a significantly reduced mucosal damage in mice treated with hBD2 (Figure 5C). Histological assessment of colon (Figure 5D) showed strong mucosal damage caused by DSS, characterized by a massive loss of the crypt architecture. In contrast, treatment with hBD2 prevented this loss of crypts and maintained a normal mucosa, comparable to the naïve mice.

### hBD2 Significantly Improved TNBS Colitis *in vivo*

**Figure 6:**
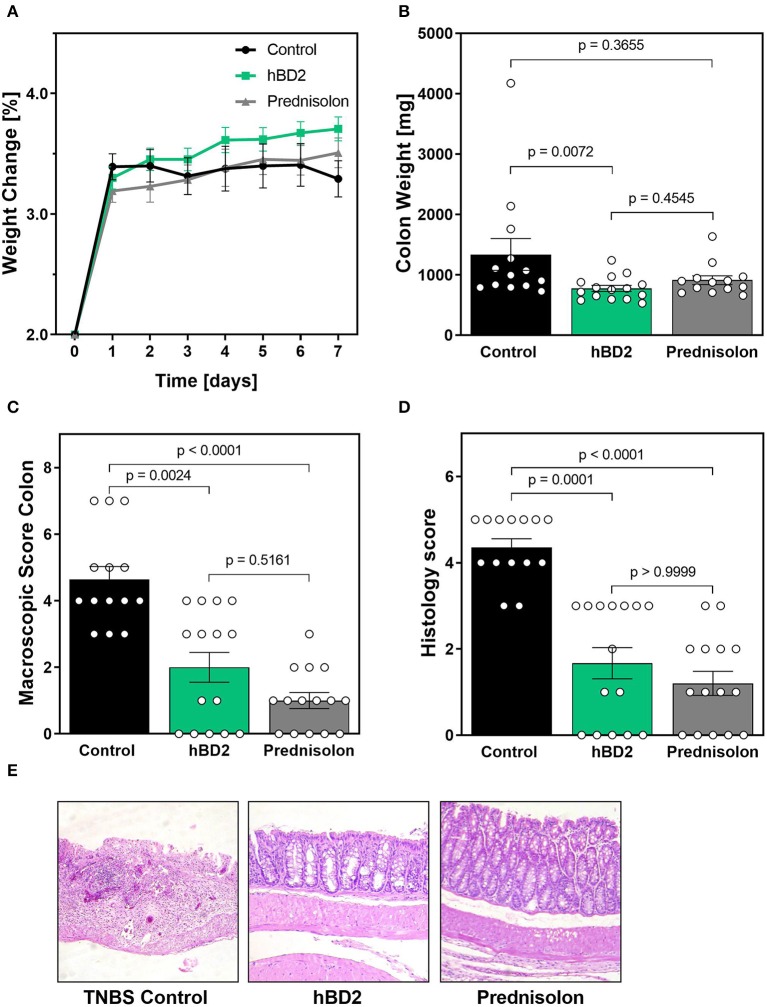
hBD2 significantly improved TNBS colitis *in vivo*. Colitis was induced by a single dose of TNBS into the colon. Mice were then treated s.c. with different doses of hBD2 (0.1 mg/kg) or Prednisolone (10 mg/kg) once a day and monitored for 7 days. **(A)** Development of mouse weight during the experiment. After euthanization, the colon of the mice was examined for **(B)** colon weight, **(C)** macroscopic abnormalities, and **(D)** microscopic evidence of inflammation. **(E)** Representative images from the colon of differentially treated mice are shown. Results are presented as mean ± SEM, control group *n* = 14, prednisolone group *n* = 14, and hBD2 group *n* = 15. Appropriate statistical comparisons are shown within the graph by a Kruskal-Wallis-test for non-parametric data with a Dunn's post-test.

We next tested the efficacy of hBD2 in TNBS induced colitis. TNBS reacts with proteins in the colon (haptenation), thus making them immunogenic. TNBS is dissolved in ethanol, which permeabilizes the colonic epithelium. The immunogenic proteins then cause a predominantly Th1 type response restricted to the colon (33). In contrast to DSS colitis, TNBS colitis did not result in weight loss, but rather prevented weight-gain in mice during the time of our experiment. Weight changes were similar between groups, although hBD2 treated mice trended toward increased weight gain from day 1–7 (Figure 6A). Yet, s.c. treatment with 0.1 mg/kg hBD2 significantly reduced colon weight and a similar tendency was also observed for prednisolone (Figure 6B), indicating a reduced infiltration of inflammatory cells. Finally, s.c. treatment with hBD2 significantly improved the macroscopic (Figure 6C) as well as the microscopic score of mouse colons (Figures 6D,E) comparable with the effect of prednisolone. Microscopic analysis of the colon showed a loss of crypts in diseased mice, while mice receiving 0.1 mg/kg hBD2 showed a nearly normal mucosa comparable to prednisolone treated mice.

### Protective Effect of hBD2 in T Cell Transfer Colitis

**Figure 7:**
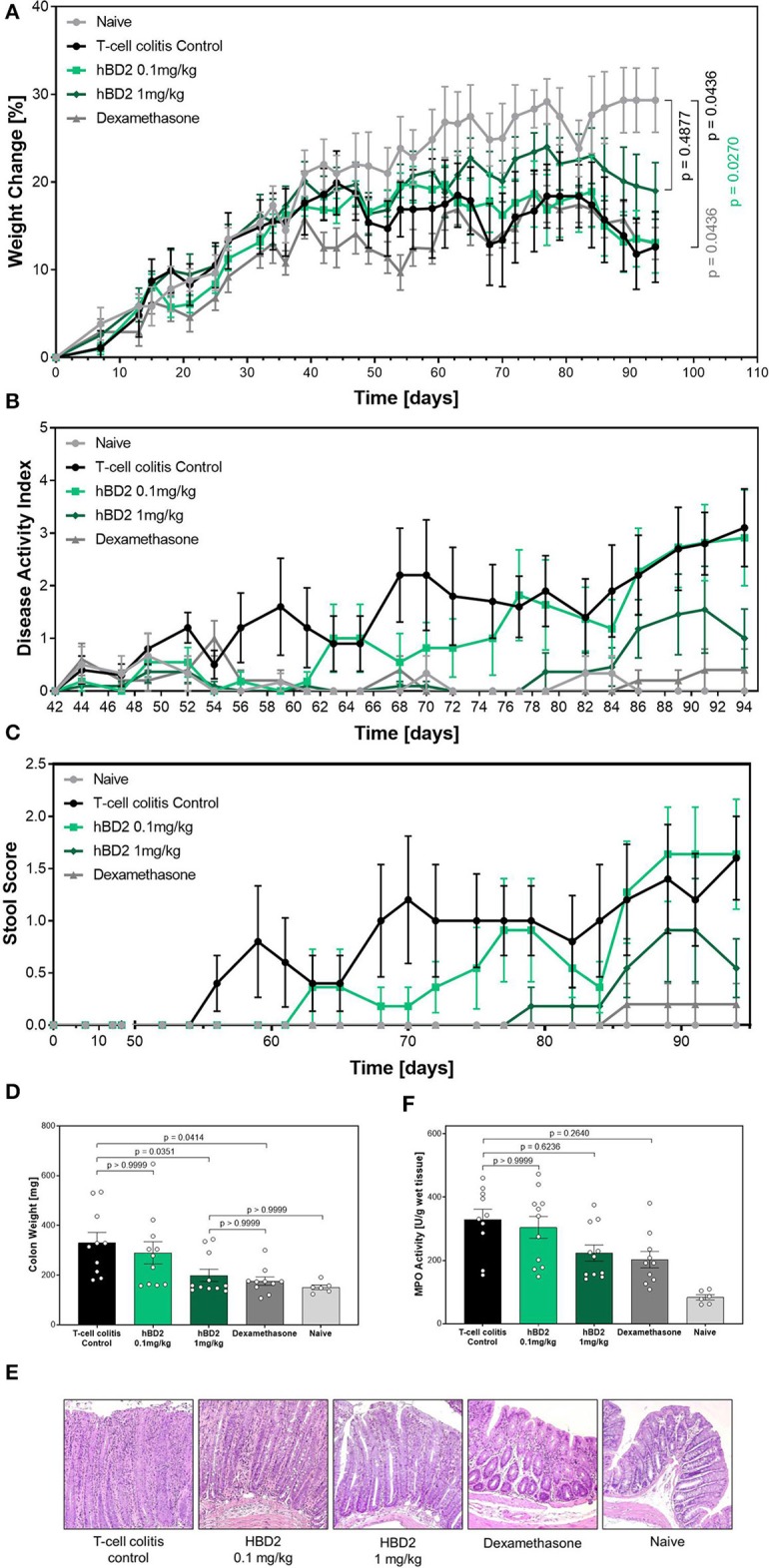
Protective effect of hBD2 in T cell transfer colitis. Colitis was induced by transferring CD4^+^/CD25^−^ T cells from WT mice to SCID mice. Development of colitis was observed for 94 days. Daily treatment with hBD2 (0.1 mg/kg or 1 mg/kg, s.c.) and Dexamethasone (0.3 mg/kg, i.p.) started 7 days after the transfer. **(A)** Weight change of mice during the experiment was monitored as well as **(B)** development of clinical symptoms as DAI. **(C)** Alteration of stool consistency was assessed as stool score. At the end of the experiment **(D)** colon weight was measured and **(E)** activity of myeloperoxidase (MPO) was quantified. **(F)** Representative images from the colon of differentially treated mice are shown. Results are presented as mean ± SEM, *n* = 6 (sham treated animals), *n* = 10 (vehicle and dexamethasone mice) and *n* = 11 (hBD2 mice). Appropriate statistical comparisons are shown within the graph by a Kruskal-Wallis-test for non-parametric data with a Dunn's post-test.

Finally, we tested hBD2 in a model of T cell transfer colitis. In this model CD4^+^ T cells from immunocompetent mice are adoptively transferred into severe combined immunodeficiency (SCID) mice, lacking T cells. The transferred T cells respond to enteric bacteria with the release of IL-2 and INF-γ (34). The inflammation is restricted to the colon, and extends diffusely from the cecum to the rectum. Besides affecting the lamina propria, the pathogenesis can also be transmural (35). Importantly, in addition to the different disease pathology, this model also results in chronic inflammation as oppose to the acute models (DSS and TNBS) previously examined. Colitis was induced in SCID mice by transferring CD4^+^/CD25^−^ T cells from WT mice. One group of SCID mice did not receive a T cell transfer (naive). Mice that received a T cell transfer gained significantly less weight than naive mice. Based on the chronic nature of this model and the involvement of numerous cell type of the immunological arsenal, we applied two different doses of hBD2 to increase the therapeutic window. Colitis mice treated with 1 mg/kg hBD2 s.c. showed less weight loss (Figure 7A) and demonstrated an improved DAI (Figure 7B) in comparison to the T cell colitis control group. Same trait was observed for the stool score (Figure 7C). The increased scores in the untreated colitis group appeared earlier than in the hBD2 treated group indicating a protective effect of hBD2. Furthermore, treatment with 1 mg/kg hBD2 significantly reduced the colon weight (Figure 7D) supporting the conclusion of mitigated inflammation. Only a minor effect of hBD2 on colonic myeloperoxidase activity could be observed (Figure 7E). Histological analysis (Figure 7F) showed pronounced inflammation in colitis mice without treatment, while mice treated with 1 mg/kg hBD2 showed less signs of inflammation and tissue disruption. Less signs of inflammation were also observed in the colon of dexamethasone treated mice. Furthermore, dexamethasone also improved DAI, stool score and colon weight significantly and was therefore superior to hBD2 in the T cell transfer model. The high dose of hBD2 (one log higher than previous experiments) seemed essential as 0.1 mg/kg hBD2 did not improve the outcome of T cell mediated colitis (Figures 7A–F).

## Discussion

Herein we report that hBD2 can be used as a systemically administered anti-inflammatory biological. hBD2 is well-tolerated, both *in vitro*, and *in vivo*. These observations are in line with published *in vitro* studies testing hBD2 with human mesenchymal stem cells, osteoblasts, keratinocytes, and HeLa cells without observing cytotoxic effects (36). Otte et al. (19) additionally found that hBD2 was well-tolerated by intestinal epithelial cells. Of note and in contrast to hBD2, high concentrations of hBD3 demonstrated cytotoxic effects in human dendritic cells and keratinocytes (37). After subcutaneous injection we observed a dose dependent pruritus of short duration which only occurred at doses magnitudes higher than the later identified therapeutic doses. At all doses (up to 40 mg/kg), body weight and organ weights of liver, spleen and kidney were unaffected.

Peak hBD2 drug serum concentrations were also dose dependent and in the range of 2–10 μg/ml after single doses of 1 vs. 10 mg/kg. Using medium concentrations in a similar range we confirmed that hBD2 attenuates inflammatory responses of human PBMC's *in vitro*. TNF-α, IL-1β, and IL-12p70 were consistently reduced after hBD2 treatment, whereas the anti-inflammatory cytokine, IL-10, was significantly increased. TNF-α is a well-known key inflammatory mediator of IBD and a successful target of modern biologicals in the treatment of IBD and other inflammatory diseases (38). In addition, IL-1β has recently been described to mediate intestinal inflammation in IBD patients with IL-10 receptor deficiency, and is thus proposed as a potential therapeutic target (39). In a follow-up pilot study using 4 colitis patients and 4 healthy controls, we confirmed the immunomodulatory capabilities of hBD2 in both groups, lending further credence to the hypothesis that hBD2 might be used as a novel biological to either treat colitis patients or alternatively to keep such patients in remission.

In a next step, we identified DCs as one cell population amongst PBMCs whose cytokine secretion is modulated by hBD2. Because it is already known that hBD2 is able to induce chemotaxis by binding to the CCR2 receptor (22), we investigated whether the observed hBD2 mediated downregulation of inflammation might depend on CCR2. In line with this hypothesis, the effects of hBD2 in human mo-DC's as well as in murine BM-DC's were completely blocked by a CCR2 inhibitor. We found a potential interaction between the downstream signaling molecules of the TLR and CCR signaling pathways. One possibility is that the signaling molecules NF-κB and CREB compete for the coactivator CBP. But also other signaling molecules such as the extracellular signal-regulated kinase (ERK) that plays an important role in TLR signaling (40) as well as CCR signaling, by inducing e.g., expression of CCR1 and CCR2 in human monocytic cells may play important roles (41, 42). Unfortunately, it is not possible to test the CCR2 dependency of the hBD2 effect *in vivo* because CCR2^−/−^ mice are protected from experimental induced colitis (43).

Based on the feasibility of recombinant hBD2 production (26), negligible toxicity, and strong anti-inflammatory CCR2 dependent modulation of DCs, we hypothesized that hBD2 could be used as a systemic anti-inflammatory biological. This strategy uncouples the classical intra-intestinal functions of hBD2 from their potent immunomodulatory capacity, and thus represents a new paradigm in therapeutic use of antimicrobial peptides. Indeed, subcutaneously administered hBD2 improved the outcome of colitis in three different *in vivo* models of IBD, namely DSS-, TNBS-, and T-cell transfer-mediated colitis. This is in line with our observation of hBD2 to rapidly enter the blood stream after s.c. administration and is likely mediated by its anti-inflammatory activity on several blood cell populations, and especially the DC fraction, as described above. We therefore tested and found that hBD2 administered s.c. could act as an immune-modulator, attenuating inflammation, that characterizes IBD. This finding is consistent with the observation of Aden et al. (44) who studied the development of colitis in IL-23 receptor deficient mice (Il23R^Δ*IEC*^) and found these to display decreased levels of leukocyte derived IL-22 and of Reg3b, a C-type lectin with antimicrobial activity (45). Systemic administration of Reg3b significantly improved DSS-colitis in Il23R^Δ*IEC*^ mice by recruiting IL-22 secreting neutrophils supporting a protective role for Reg3b in colitis (44). In addition, the hBD2-induced wound healing in intestinal epithelial cells *in vitro* (19) may add another mechanism of action. Finally, rectally applied porcine β-defensin 2 (pBD2) has been used for the treatment of DSS colitis in mice by Han et al. (46). They found pBD2 to be protective against mucosal injury and disruption of the epithelial barrier associated with DSS colitis. Furthermore, they reported decreased inflammatory infiltrates and expression of inflammatory mediators upon treatment with pBD2 as well as an increase in intestinal tight junction structure and function compared to untreated DSS control mice. In contrast to the local administration employed by Han et al., we provide evidence for a distinct anti-inflammatory effect with systemic application.

Despite ongoing development of therapeutic approaches, new treatment strategies for the management of IBD are still urgently needed. Corticosteroids remain the standard therapeutic options for active CD and UC. However, their beneficial effects are associated with severe side effects such as osteoporosis, moon face, mood disturbances, glaucoma, and hypertension (47). In recent years, several new therapeutics targeting the molecular mechanisms of intestinal and systemic inflammation have been developed. Among these, anti-TNFα antibodies have been the most successful and most commonly used biological (48). However, primary and secondary lack of response as well as serious side effects, limits their use. More recently, antibodies targeting IL-17 in CD and IL-13 in UC have been proposed as IBD management. Yet, despite promising preclinical data, both antibodies failed to improve the outcome of CD or UC (49) in patients. Secukinumab, an anti-IL-17 antibody, even worsened the disease in a clinical trial in CD patients (50) and two anti-IL-13 antibodies also failed to produce positive results in clinical trials for UC (51, 52). This negative effect of IL-17 blockade may in part be explained by blocking the hBD2 pathway, which is also mediated by IL-17.

In conclusion, the results presented here constitute the first *in vivo* proof of therapeutic efficacy of a systemically administered human defensin. It is, however, important to stress, that in all tested models, hBD2 were administered before the onset of clinical inflammation. We therefore provide strong evidence for the potential of hBD2 treatment to keep patients in remission, but studies are warranted to examine if hBD2 also exhibits treatment efficacy of acute and relapsed inflammation. The use of natural host defense peptides could provide a new chapter of effective, minimal-side-effect treatment strategies of IBD.

## Data Availability Statement

The datasets generated for this study are available on request to the corresponding author.

## Ethics Statement

The studies involving human participants were reviewed and approved by the Ethical Committee of the University Hospital Tübingen. The patients/participants provided their written informed consent to participate in this study. The animal study was reviewed and approved by the Novozymes science ethics committee.

## Author Contributions

LK, NA, KB, SK, BA, PN, BJ, and JW conceived the study, designed the experiments, and analyzed the data. LK, NA, KB, BA, and CL performed the experiments. SA, DS, and NM contributed reagents, materials, analysis tools, and participated in scientific discussion. LK, NA, ES, PN, BJ, and JW wrote the paper.

### Conflict of Interest

KB, SK, and BA was employed by Novozymes. PN was employed by Defensin Therapeutics. PN and JW hold shares of Defensin Therapeutics. Defensin Therapeutics holds patents on treatment with defensins. The remaining authors declare that the research was conducted in the absence of any commercial or financial relationships that could be construed as a potential conflict of interest.
