# Supplementary material for: Human β-Defensin 2 Mediated Immune Modulation as Treatment for Experimental Colitis
Source: Front Immunol. 2020 Jan 31;11:93. doi: 10.3389/fimmu.2020.00093 (PMC7006816; doi:10.3389/fimmu.2020.00093)
Supplement: Supplementary file 1 [file Data_Sheet_1.PDF]

Supplementary Material

Supplementary Figure 1: TLR- and CCR2-dependent cytokine production of DC´s affected by hBD2

Human mo-DC´s were treated with LPS (10 µg/ml) alone or co-incubated with various concentrations of hBD2 (100 µg/ml or 10 µg/ml) and additionally pre-treated with CCR2 inhibitor RS prior to stimulation. (B-G) BM-DCs were incubated with LPS or a cytokine cocktail containing TNF-α (0.2 mg/ml), IL-6 (0.2 mg/ml) and IL-1β (0.2 mg/ml) and additionally co-incubated with hBD2 (100 µg/ml). BM-DCs were additionally pre-treated with pertussis toxin or the CCR2 inhibitor RS. Release of TNF-α in human Mo-DC´s (A) was quantified by ELISA. Release of IL-12p70 and IL-1β in murine BM-DC´s (B-G) was quantified by LEGENDplex. Results are presented as mean ± SEM, n = 3. Statistical test used is one-way ANOVA with Bonferroni post-test.

Supplementary Figure 2: Lack of toxicity *in vitro* and *in vivo* and favorable pharmacokinetics of recombinant hBD2 after subcutaneous administration

Different mammalian cell types were treated with various concentrations of hBD2. (A) Hemolytic effect of hBD2 in human red blood cells relative to negative control. n = 1. SDS was used as a positive control. (B) Viability of murine fibroblasts in presence of hBD2. n = 1. SDS was used as a positive control. (C-E) Mice were injected s.c. with different doses of hBD2. (C) Body weight gain of mice from day 0-4 after s.c. injection of hBD2, individual weights of mice are shown and (D) the weight of liver, spleen and kidneys taken from mice during necropsy (control mice n = 2 and hBD2 mice n = 3). (E) Quantification of hBD2 in the serum of mice after s.c. application (1 mg/kg n = 4 and 10 mg/kg n = 3). Results of (D) and (E) are shown as mean ± SEM.
