# Supplementary figures and images for: Human β-Defensin 2 Mediated Immune Modulation as Treatment for Experimental Colitis
Source: Front Immunol. 2020 Jan 31;11:93. doi: 10.3389/fimmu.2020.00093 (PMC7006816; doi:10.3389/fimmu.2020.00093)

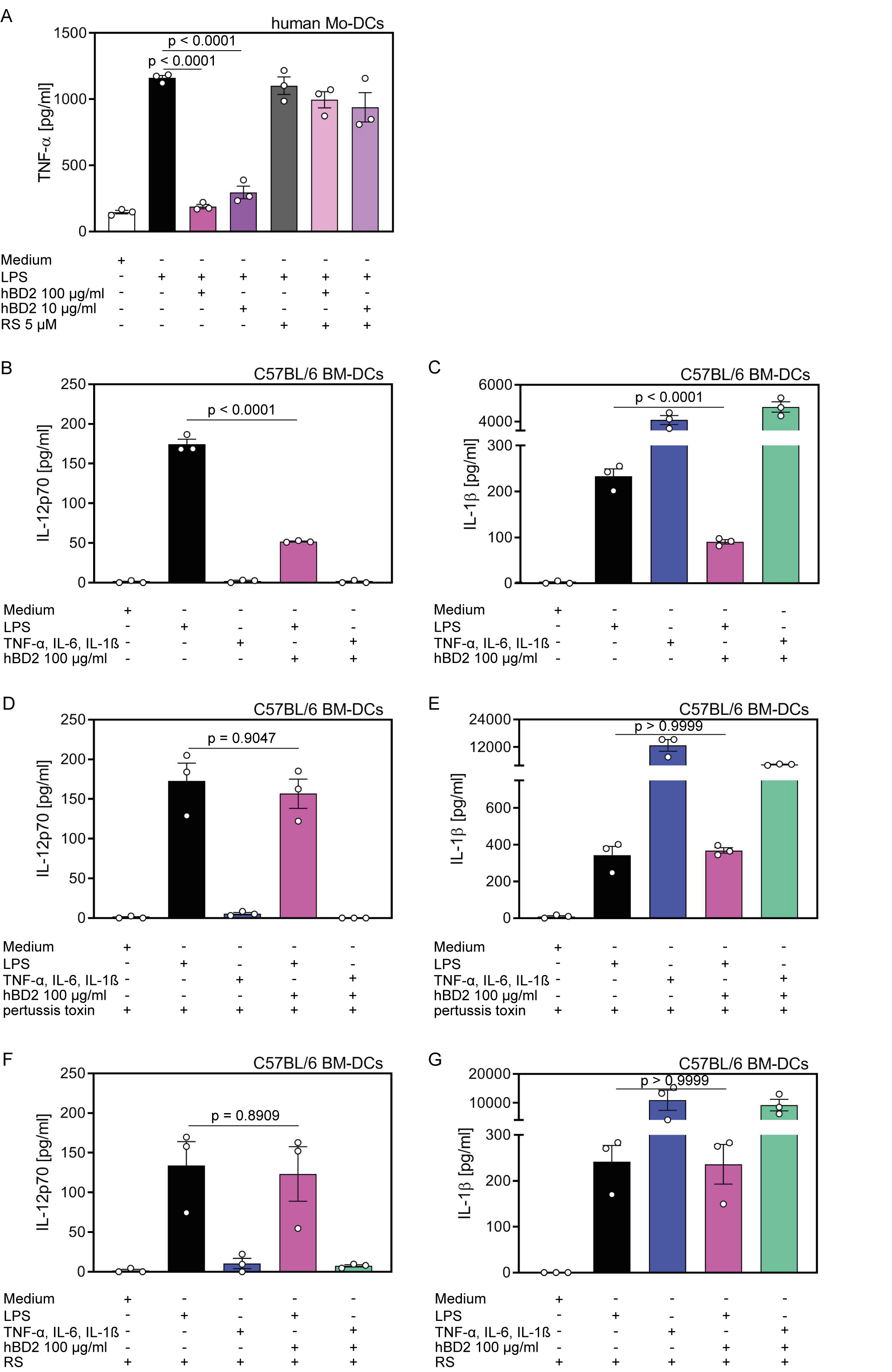

Supplement: Supplementary file 2 [file Image_1.JPEG]

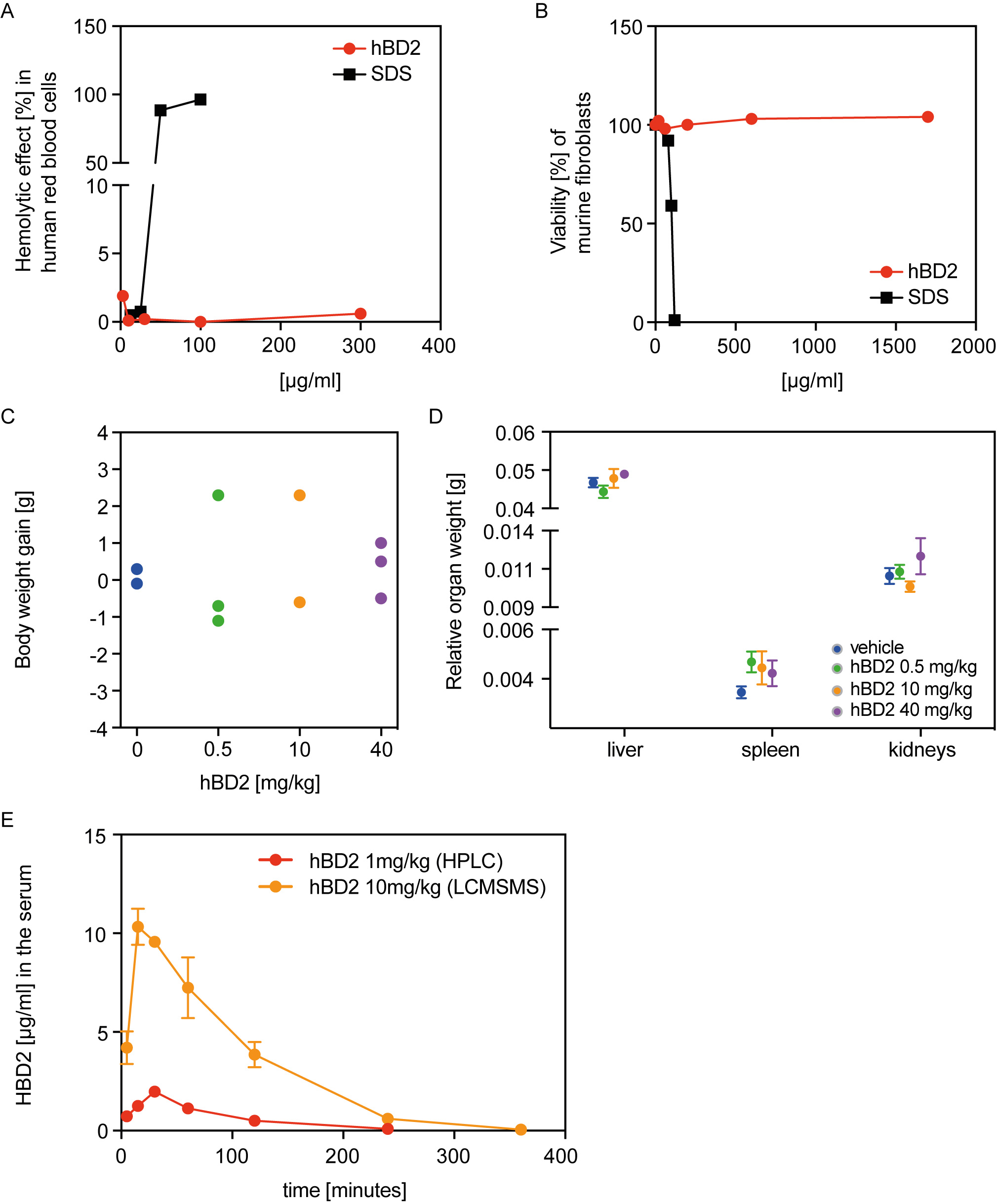

Supplement: Supplementary file 3 [file Image_2.JPEG]
